# Supplementary material for: Modeling glioblastoma heterogeneity as a dynamic network of cell states
Source: Mol Syst Biol. 2021 Sep 16;17(9):e10105. doi: 10.15252/msb.202010105 (PMC8444284; doi:10.15252/msb.202010105)
Supplement: Supplementary file 6 — Source Data for Figure 5 [file MSB-17-e10105-s004.zip › Figure5A_sourcedata/GSEA_3017/nosigdb_stateF.GseaPreranked.1622795015644/DOROSZKO_U3054MG_CLUSTER_4.html]

Details for gene set DOROSZKO\_U3054MG\_CLUSTER\_4[GSEA]

|  || Dataset | state33017 |
| Phenotype | NoPhenotypeAvailable |
| Upregulated in class | na\_neg |
| GeneSet | DOROSZKO\_U3054MG\_CLUSTER\_4 |
| Enrichment Score (ES) | -0.755406 |
| Normalized Enrichment Score (NES) | -6.066912 |
| Nominal p-value | 0.0 |
| FDR q-value | 0.0 |
| FWER p-Value | 0.0 |
Table: GSEA Results Summary

  

Fig 1: Enrichment plot: DOROSZKO\_U3054MG\_CLUSTER\_4      
 Profile of the Running ES Score & Positions of GeneSet Members on the Rank Ordered List

  

| PROBE | GENE SYMBOL | GENE\_TITLE | RANK IN GENE LIST | RANK METRIC SCORE | RUNNING ES | CORE ENRICHMENT || 1 | DDB2 |  |  | 143 | 0.682 | -0.0709 | No |
| 2 | TCEAL7 |  |  | 416 | 0.464 | -0.2098 | No |
| 3 | DRAXIN |  |  | 428 | 0.460 | -0.2132 | No |
| 4 | PMP2 |  |  | 562 | 0.411 | -0.2802 | No |
| 5 | IKBIP |  |  | 567 | 0.408 | -0.2803 | No |
| 6 | PPP1R14C |  |  | 733 | 0.363 | -0.3641 | No |
| 7 | PDLIM4 |  |  | 982 | 0.313 | -0.4913 | No |
| 8 | RTN3 |  |  | 1095 | 0.293 | -0.5480 | No |
| 9 | ALDH7A1 |  |  | 1255 | 0.273 | -0.6292 | No |
| 10 | AEN |  |  | 1260 | 0.273 | -0.6299 | No |
| 11 | PSMB9 |  |  | 1347 | 0.262 | -0.6733 | No |
| 12 | ABHD3 |  |  | 1382 | 0.259 | -0.6897 | No |
| 13 | SDC1 |  |  | 1438 | 0.254 | -0.7169 | No |
| 14 | FANCL |  |  | 1442 | 0.253 | -0.7172 | No |
| 15 | RFC5 |  |  | 1473 | -0.250 | -0.7316 | No |
| 16 | ERCC6L |  |  | 1481 | -0.252 | -0.7340 | No |
| 17 | RPA2 |  |  | 1490 | -0.256 | -0.7368 | No |
| 18 | NCAPH2 |  |  | 1496 | -0.258 | -0.7381 | No |
| 19 | CBFB |  |  | 1499 | -0.259 | -0.7379 | No |
| 20 | BLM |  |  | 1507 | -0.262 | -0.7402 | No |
| 21 | ACAT2 |  |  | 1512 | -0.263 | -0.7410 | No |
| 22 | MSH6 |  |  | 1517 | -0.264 | -0.7418 | No |
| 23 | RBBP8 |  |  | 1518 | -0.265 | -0.7404 | No |
| 24 | PPP2R3B |  |  | 1520 | -0.265 | -0.7396 | No |
| 25 | CEP78 |  |  | 1524 | -0.265 | -0.7399 | No |
| 26 | PHTF2 |  |  | 1529 | -0.267 | -0.7406 | No |
| 27 | PSMD1 |  |  | 1532 | -0.267 | -0.7403 | No |
| 28 | MAD2L2 |  |  | 1539 | -0.269 | -0.7421 | No |
| 29 | CCDC138 |  |  | 1541 | -0.270 | -0.7413 | No |
| 30 | MCMBP |  |  | 1546 | -0.271 | -0.7420 | No |
| 31 | BAZ1A |  |  | 1547 | -0.271 | -0.7407 | No |
| 32 | NDUFAF2 |  |  | 1557 | -0.274 | -0.7440 | No |
| 33 | NUP62 |  |  | 1562 | -0.275 | -0.7447 | No |
| 34 | PSIP1 |  |  | 1564 | -0.275 | -0.7438 | No |
| 35 | NAP1L4 |  |  | 1568 | -0.277 | -0.7440 | No |
| 36 | WDHD1 |  |  | 1569 | -0.277 | -0.7426 | No |
| 37 | PAXX |  |  | 1575 | -0.279 | -0.7439 | No |
| 38 | CD320 |  |  | 1577 | -0.279 | -0.7430 | No |
| 39 | PPIH |  |  | 1579 | -0.281 | -0.7421 | No |
| 40 | E2F1 |  |  | 1580 | -0.281 | -0.7407 | No |
| 41 | FKBP5 |  |  | 1581 | -0.281 | -0.7393 | No |
| 42 | CDKN2C |  |  | 1593 | -0.285 | -0.7436 | No |
| 43 | LHX2 |  |  | 1597 | -0.285 | -0.7437 | No |
| 44 | RCC1 |  |  | 1604 | -0.286 | -0.7454 | No |
| 45 | RAD1 |  |  | 1613 | -0.289 | -0.7482 | No |
| 46 | CENPX |  |  | 1621 | -0.291 | -0.7503 | No |
| 47 | POLA2 |  |  | 1622 | -0.291 | -0.7489 | No |
| 48 | UFD1 |  |  | 1628 | -0.293 | -0.7500 | No |
| 49 | TMEM97 |  |  | 1629 | -0.293 | -0.7486 | No |
| 50 | HES6 |  |  | 1632 | -0.294 | -0.7481 | No |
| 51 | RSRC1 |  |  | 1633 | -0.294 | -0.7467 | No |
| 52 | GINS1 |  |  | 1634 | -0.295 | -0.7452 | No |
| 53 | HDGFL3 |  |  | 1635 | -0.295 | -0.7437 | No |
| 54 | SNRPA |  |  | 1637 | -0.296 | -0.7428 | No |
| 55 | CENPS |  |  | 1646 | -0.299 | -0.7455 | No |
| 56 | PARP2 |  |  | 1665 | -0.305 | -0.7533 | No |
| 57 | FAM136A |  |  | 1666 | -0.306 | -0.7518 | No |
| 58 | KLHL23 |  |  | 1674 | -0.308 | -0.7539 | Yes |
| 59 | CEP57 |  |  | 1676 | -0.309 | -0.7529 | Yes |
| 60 | EIF4EBP1 |  |  | 1681 | -0.312 | -0.7534 | Yes |
| 61 | FADS1 |  |  | 1682 | -0.312 | -0.7518 | Yes |
| 62 | NUP107 |  |  | 1683 | -0.313 | -0.7503 | Yes |
| 63 | DCXR |  |  | 1689 | -0.315 | -0.7513 | Yes |
| 64 | CTPS1 |  |  | 1692 | -0.316 | -0.7508 | Yes |
| 65 | MRE11 |  |  | 1693 | -0.316 | -0.7492 | Yes |
| 66 | MAP3K20 |  |  | 1694 | -0.317 | -0.7476 | Yes |
| 67 | MGME1 |  |  | 1697 | -0.317 | -0.7471 | Yes |
| 68 | ZNF93 |  |  | 1699 | -0.318 | -0.7460 | Yes |
| 69 | PRIM1 |  |  | 1703 | -0.320 | -0.7460 | Yes |
| 70 | HAUS1 |  |  | 1708 | -0.322 | -0.7465 | Yes |
| 71 | TRIM28 |  |  | 1709 | -0.322 | -0.7449 | Yes |
| 72 | CENPQ |  |  | 1711 | -0.322 | -0.7438 | Yes |
| 73 | MRPL14 |  |  | 1714 | -0.323 | -0.7432 | Yes |
| 74 | SASS6 |  |  | 1719 | -0.325 | -0.7437 | Yes |
| 75 | MCM3 |  |  | 1738 | -0.335 | -0.7513 | Yes |
| 76 | GPN3 |  |  | 1739 | -0.335 | -0.7497 | Yes |
| 77 | PLK4 |  |  | 1740 | -0.336 | -0.7480 | Yes |
| 78 | RMI1 |  |  | 1743 | -0.337 | -0.7474 | Yes |
| 79 | STIL |  |  | 1744 | -0.337 | -0.7457 | Yes |
| 80 | EXOSC9 |  |  | 1746 | -0.338 | -0.7445 | Yes |
| 81 | COA1 |  |  | 1753 | -0.341 | -0.7460 | Yes |
| 82 | GLO1 |  |  | 1755 | -0.342 | -0.7448 | Yes |
| 83 | PAICS |  |  | 1757 | -0.342 | -0.7436 | Yes |
| 84 | PPM1G |  |  | 1771 | -0.348 | -0.7486 | Yes |
| 85 | SLF1 |  |  | 1773 | -0.350 | -0.7474 | Yes |
| 86 | RRP7A |  |  | 1775 | -0.351 | -0.7462 | Yes |
| 87 | TOPBP1 |  |  | 1781 | -0.354 | -0.7470 | Yes |
| 88 | RABL6 |  |  | 1782 | -0.354 | -0.7452 | Yes |
| 89 | WDR76 |  |  | 1783 | -0.355 | -0.7435 | Yes |
| 90 | SRPK1 |  |  | 1788 | -0.356 | -0.7438 | Yes |
| 91 | BRIP1 |  |  | 1790 | -0.357 | -0.7425 | Yes |
| 92 | POC1A |  |  | 1794 | -0.360 | -0.7423 | Yes |
| 93 | LIG1 |  |  | 1797 | -0.361 | -0.7415 | Yes |
| 94 | CENPL |  |  | 1805 | -0.362 | -0.7434 | Yes |
| 95 | WDR34 |  |  | 1806 | -0.365 | -0.7415 | Yes |
| 96 | NUP50 |  |  | 1823 | -0.376 | -0.7480 | Yes |
| 97 | MRPL12 |  |  | 1831 | -0.380 | -0.7497 | Yes |
| 98 | C12orf75 |  |  | 1832 | -0.380 | -0.7478 | Yes |
| 99 | CDC45 |  |  | 1836 | -0.382 | -0.7475 | Yes |
| 100 | BARD1 |  |  | 1839 | -0.384 | -0.7466 | Yes |
| 101 | ZNF519 |  |  | 1841 | -0.385 | -0.7452 | Yes |
| 102 | MASTL |  |  | 1843 | -0.385 | -0.7438 | Yes |
| 103 | FANCA |  |  | 1844 | -0.385 | -0.7419 | Yes |
| 104 | FBL |  |  | 1845 | -0.386 | -0.7400 | Yes |
| 105 | MMS22L |  |  | 1846 | -0.387 | -0.7381 | Yes |
| 106 | SMCHD1 |  |  | 1847 | -0.387 | -0.7361 | Yes |
| 107 | SVIP |  |  | 1849 | -0.388 | -0.7347 | Yes |
| 108 | SRSF10 |  |  | 1851 | -0.390 | -0.7333 | Yes |
| 109 | CDCA4 |  |  | 1852 | -0.391 | -0.7314 | Yes |
| 110 | PCNT |  |  | 1854 | -0.392 | -0.7299 | Yes |
| 111 | ACTL6A |  |  | 1859 | -0.393 | -0.7301 | Yes |
| 112 | KIF18B |  |  | 1860 | -0.393 | -0.7281 | Yes |
| 113 | FANCB |  |  | 1862 | -0.396 | -0.7267 | Yes |
| 114 | E2F8 |  |  | 1864 | -0.396 | -0.7252 | Yes |
| 115 | EXOSC8 |  |  | 1865 | -0.397 | -0.7232 | Yes |
| 116 | PKMYT1 |  |  | 1876 | -0.402 | -0.7264 | Yes |
| 117 | TEDC1 |  |  | 1883 | -0.410 | -0.7275 | Yes |
| 118 | CENPO |  |  | 1884 | -0.410 | -0.7255 | Yes |
| 119 | SHCBP1 |  |  | 1886 | -0.412 | -0.7239 | Yes |
| 120 | RFC4 |  |  | 1887 | -0.413 | -0.7219 | Yes |
| 121 | POLQ |  |  | 1891 | -0.417 | -0.7214 | Yes |
| 122 | SAE1 |  |  | 1893 | -0.417 | -0.7198 | Yes |
| 123 | ASF1B |  |  | 1897 | -0.419 | -0.7193 | Yes |
| 124 | RAD18 |  |  | 1898 | -0.419 | -0.7172 | Yes |
| 125 | NSD2 |  |  | 1900 | -0.425 | -0.7156 | Yes |
| 126 | POLE3 |  |  | 1901 | -0.426 | -0.7135 | Yes |
| 127 | DKC1 |  |  | 1904 | -0.428 | -0.7124 | Yes |
| 128 | MXD3 |  |  | 1905 | -0.428 | -0.7103 | Yes |
| 129 | MNS1 |  |  | 1913 | -0.430 | -0.7117 | Yes |
| 130 | HMGA1 |  |  | 1915 | -0.433 | -0.7101 | Yes |
| 131 | TTF1 |  |  | 1917 | -0.436 | -0.7085 | Yes |
| 132 | POLD3 |  |  | 1918 | -0.436 | -0.7063 | Yes |
| 133 | UHRF1 |  |  | 1921 | -0.438 | -0.7052 | Yes |
| 134 | DIAPH3 |  |  | 1924 | -0.439 | -0.7040 | Yes |
| 135 | HMGXB4 |  |  | 1928 | -0.441 | -0.7034 | Yes |
| 136 | GGCT |  |  | 1930 | -0.442 | -0.7017 | Yes |
| 137 | GINS2 |  |  | 1932 | -0.445 | -0.7000 | Yes |
| 138 | STIP1 |  |  | 1933 | -0.446 | -0.6978 | Yes |
| 139 | MZT1 |  |  | 1936 | -0.447 | -0.6966 | Yes |
| 140 | TTF2 |  |  | 1942 | -0.449 | -0.6970 | Yes |
| 141 | TPRKB |  |  | 1944 | -0.450 | -0.6952 | Yes |
| 142 | LRRC59 |  |  | 1945 | -0.450 | -0.6930 | Yes |
| 143 | DDX11 |  |  | 1952 | -0.456 | -0.6938 | Yes |
| 144 | NOP56 |  |  | 1953 | -0.456 | -0.6916 | Yes |
| 145 | RFC2 |  |  | 1954 | -0.457 | -0.6893 | Yes |
| 146 | DDX39A |  |  | 1957 | -0.458 | -0.6881 | Yes |
| 147 | DDIAS |  |  | 1960 | -0.460 | -0.6868 | Yes |
| 148 | CDT1 |  |  | 1961 | -0.460 | -0.6845 | Yes |
| 149 | ZWILCH |  |  | 1962 | -0.461 | -0.6822 | Yes |
| 150 | DNMT1 |  |  | 1969 | -0.465 | -0.6830 | Yes |
| 151 | TIMM10 |  |  | 1975 | -0.470 | -0.6833 | Yes |
| 152 | CMC2 |  |  | 1977 | -0.473 | -0.6814 | Yes |
| 153 | C19orf48 |  |  | 1979 | -0.474 | -0.6796 | Yes |
| 154 | POP7 |  |  | 1981 | -0.474 | -0.6778 | Yes |
| 155 | CSE1L |  |  | 1985 | -0.479 | -0.6769 | Yes |
| 156 | SUPT16H |  |  | 1986 | -0.479 | -0.6746 | Yes |
| 157 | NASP |  |  | 1988 | -0.481 | -0.6727 | Yes |
| 158 | TIMELESS |  |  | 1989 | -0.482 | -0.6703 | Yes |
| 159 | PGP |  |  | 1990 | -0.482 | -0.6679 | Yes |
| 160 | CDC6 |  |  | 1991 | -0.483 | -0.6655 | Yes |
| 161 | PHIP |  |  | 1992 | -0.483 | -0.6631 | Yes |
| 162 | MCM4 |  |  | 1993 | -0.487 | -0.6607 | Yes |
| 163 | SPAG5 |  |  | 1997 | -0.490 | -0.6598 | Yes |
| 164 | PSMC3 |  |  | 1999 | -0.492 | -0.6579 | Yes |
| 165 | FAM111A |  |  | 2002 | -0.497 | -0.6564 | Yes |
| 166 | TK1 |  |  | 2003 | -0.497 | -0.6540 | Yes |
| 167 | LRR1 |  |  | 2004 | -0.497 | -0.6515 | Yes |
| 168 | TUBB6 |  |  | 2006 | -0.500 | -0.6495 | Yes |
| 169 | XPO1 |  |  | 2007 | -0.500 | -0.6470 | Yes |
| 170 | TOMM40 |  |  | 2008 | -0.501 | -0.6445 | Yes |
| 171 | CDK5RAP2 |  |  | 2012 | -0.503 | -0.6436 | Yes |
| 172 | CHEK1 |  |  | 2013 | -0.504 | -0.6411 | Yes |
| 173 | MAGOHB |  |  | 2015 | -0.505 | -0.6391 | Yes |
| 174 | HIRIP3 |  |  | 2017 | -0.508 | -0.6371 | Yes |
| 175 | FEN1 |  |  | 2018 | -0.511 | -0.6345 | Yes |
| 176 | SMC1A |  |  | 2021 | -0.513 | -0.6330 | Yes |
| 177 | RRM1 |  |  | 2023 | -0.516 | -0.6310 | Yes |
| 178 | SRRT |  |  | 2025 | -0.517 | -0.6289 | Yes |
| 179 | HAUS8 |  |  | 2026 | -0.517 | -0.6263 | Yes |
| 180 | CACYBP |  |  | 2028 | -0.518 | -0.6243 | Yes |
| 181 | PARPBP |  |  | 2030 | -0.520 | -0.6222 | Yes |
| 182 | JPT1 |  |  | 2036 | -0.525 | -0.6222 | Yes |
| 183 | HELLS |  |  | 2038 | -0.528 | -0.6201 | Yes |
| 184 | SNRPA1 |  |  | 2040 | -0.528 | -0.6180 | Yes |
| 185 | CDCA3 |  |  | 2041 | -0.529 | -0.6154 | Yes |
| 186 | TRIP13 |  |  | 2044 | -0.530 | -0.6138 | Yes |
| 187 | NCAPD3 |  |  | 2051 | -0.537 | -0.6142 | Yes |
| 188 | KIF22 |  |  | 2053 | -0.541 | -0.6120 | Yes |
| 189 | CENPJ |  |  | 2054 | -0.544 | -0.6093 | Yes |
| 190 | CKLF |  |  | 2055 | -0.544 | -0.6066 | Yes |
| 191 | SNRPF |  |  | 2057 | -0.545 | -0.6044 | Yes |
| 192 | CTNNAL1 |  |  | 2059 | -0.548 | -0.6022 | Yes |
| 193 | SAMD1 |  |  | 2060 | -0.549 | -0.5995 | Yes |
| 194 | SPDL1 |  |  | 2064 | -0.569 | -0.5982 | Yes |
| 195 | DTL |  |  | 2066 | -0.573 | -0.5959 | Yes |
| 196 | FANCD2 |  |  | 2068 | -0.577 | -0.5935 | Yes |
| 197 | ZWINT |  |  | 2069 | -0.577 | -0.5907 | Yes |
| 198 | CEP152 |  |  | 2070 | -0.578 | -0.5878 | Yes |
| 199 | RTKN2 |  |  | 2071 | -0.579 | -0.5849 | Yes |
| 200 | RACGAP1 |  |  | 2072 | -0.579 | -0.5820 | Yes |
| 201 | MND1 |  |  | 2074 | -0.586 | -0.5796 | Yes |
| 202 | GINS4 |  |  | 2075 | -0.586 | -0.5767 | Yes |
| 203 | LSM4 |  |  | 2076 | -0.587 | -0.5738 | Yes |
| 204 | SKA3 |  |  | 2077 | -0.590 | -0.5709 | Yes |
| 205 | BUB3 |  |  | 2078 | -0.596 | -0.5679 | Yes |
| 206 | AURKB |  |  | 2079 | -0.598 | -0.5649 | Yes |
| 207 | GGH |  |  | 2080 | -0.603 | -0.5619 | Yes |
| 208 | CENPK |  |  | 2082 | -0.607 | -0.5594 | Yes |
| 209 | USP1 |  |  | 2084 | -0.612 | -0.5569 | Yes |
| 210 | NCAPD2 |  |  | 2089 | -0.627 | -0.5558 | Yes |
| 211 | SSRP1 |  |  | 2091 | -0.630 | -0.5532 | Yes |
| 212 | MCM8 |  |  | 2092 | -0.635 | -0.5501 | Yes |
| 213 | FAM111B |  |  | 2094 | -0.637 | -0.5474 | Yes |
| 214 | MIS18A |  |  | 2096 | -0.639 | -0.5448 | Yes |
| 215 | DNAJC9 |  |  | 2097 | -0.641 | -0.5416 | Yes |
| 216 | RFC3 |  |  | 2098 | -0.642 | -0.5384 | Yes |
| 217 | BRCA2 |  |  | 2100 | -0.650 | -0.5357 | Yes |
| 218 | PHF19 |  |  | 2101 | -0.650 | -0.5324 | Yes |
| 219 | ITGB3BP |  |  | 2102 | -0.652 | -0.5292 | Yes |
| 220 | NCAPH |  |  | 2104 | -0.652 | -0.5265 | Yes |
| 221 | PSMC3IP |  |  | 2106 | -0.659 | -0.5237 | Yes |
| 222 | CKAP2L |  |  | 2109 | -0.663 | -0.5214 | Yes |
| 223 | ALYREF |  |  | 2111 | -0.677 | -0.5186 | Yes |
| 224 | RPA3 |  |  | 2112 | -0.678 | -0.5152 | Yes |
| 225 | CKS2 |  |  | 2115 | -0.694 | -0.5128 | Yes |
| 226 | SRSF2 |  |  | 2122 | -0.706 | -0.5124 | Yes |
| 227 | CENPM |  |  | 2125 | -0.709 | -0.5099 | Yes |
| 228 | RAD51AP1 |  |  | 2127 | -0.715 | -0.5069 | Yes |
| 229 | CDCA8 |  |  | 2129 | -0.722 | -0.5038 | Yes |
| 230 | CKAP2 |  |  | 2131 | -0.729 | -0.5007 | Yes |
| 231 | CIP2A |  |  | 2132 | -0.733 | -0.4971 | Yes |
| 232 | ATAD5 |  |  | 2133 | -0.742 | -0.4934 | Yes |
| 233 | VRK1 |  |  | 2134 | -0.743 | -0.4897 | Yes |
| 234 | TUBA1C |  |  | 2135 | -0.756 | -0.4859 | Yes |
| 235 | KIF2C |  |  | 2136 | -0.756 | -0.4821 | Yes |
| 236 | BRCA1 |  |  | 2138 | -0.758 | -0.4789 | Yes |
| 237 | CMSS1 |  |  | 2140 | -0.772 | -0.4756 | Yes |
| 238 | KIF14 |  |  | 2141 | -0.773 | -0.4717 | Yes |
| 239 | NDC80 |  |  | 2142 | -0.775 | -0.4679 | Yes |
| 240 | FBXO5 |  |  | 2144 | -0.779 | -0.4645 | Yes |
| 241 | KIF23 |  |  | 2145 | -0.782 | -0.4606 | Yes |
| 242 | GMNN |  |  | 2146 | -0.785 | -0.4567 | Yes |
| 243 | LMNB2 |  |  | 2147 | -0.786 | -0.4528 | Yes |
| 244 | MELK |  |  | 2148 | -0.786 | -0.4489 | Yes |
| 245 | ANLN |  |  | 2149 | -0.797 | -0.4449 | Yes |
| 246 | SMC3 |  |  | 2150 | -0.800 | -0.4410 | Yes |
| 247 | KIFC1 |  |  | 2151 | -0.802 | -0.4370 | Yes |
| 248 | DEPDC1 |  |  | 2152 | -0.809 | -0.4329 | Yes |
| 249 | NCAPG2 |  |  | 2156 | -0.827 | -0.4304 | Yes |
| 250 | MCM7 |  |  | 2160 | -0.839 | -0.4278 | Yes |
| 251 | ECT2 |  |  | 2163 | -0.846 | -0.4246 | Yes |
| 252 | HMGB3 |  |  | 2164 | -0.852 | -0.4204 | Yes |
| 253 | KIF20B |  |  | 2165 | -0.852 | -0.4161 | Yes |
| 254 | SPC24 |  |  | 2166 | -0.853 | -0.4119 | Yes |
| 255 | ARHGAP11A |  |  | 2167 | -0.855 | -0.4076 | Yes |
| 256 | DTYMK |  |  | 2168 | -0.858 | -0.4034 | Yes |
| 257 | MCM10 |  |  | 2169 | -0.858 | -0.3991 | Yes |
| 258 | BUB1 |  |  | 2170 | -0.860 | -0.3948 | Yes |
| 259 | FOXM1 |  |  | 2171 | -0.861 | -0.3905 | Yes |
| 260 | CDCA5 |  |  | 2172 | -0.866 | -0.3862 | Yes |
| 261 | BUB1B |  |  | 2174 | -0.871 | -0.3824 | Yes |
| 262 | PIMREG |  |  | 2175 | -0.876 | -0.3781 | Yes |
| 263 | NUF2 |  |  | 2176 | -0.877 | -0.3737 | Yes |
| 264 | CEP55 |  |  | 2177 | -0.883 | -0.3693 | Yes |
| 265 | KPNA2 |  |  | 2178 | -0.894 | -0.3649 | Yes |
| 266 | HMMR |  |  | 2179 | -0.895 | -0.3604 | Yes |
| 267 | CCDC34 |  |  | 2180 | -0.898 | -0.3559 | Yes |
| 268 | CCNA2 |  |  | 2181 | -0.902 | -0.3514 | Yes |
| 269 | CHAF1A |  |  | 2182 | -0.903 | -0.3470 | Yes |
| 270 | MIS18BP1 |  |  | 2183 | -0.903 | -0.3425 | Yes |
| 271 | SMS |  |  | 2184 | -0.910 | -0.3379 | Yes |
| 272 | KIF15 |  |  | 2185 | -0.915 | -0.3334 | Yes |
| 273 | KIF4A |  |  | 2186 | -0.921 | -0.3288 | Yes |
| 274 | PRR11 |  |  | 2187 | -0.930 | -0.3242 | Yes |
| 275 | ORC6 |  |  | 2188 | -0.937 | -0.3195 | Yes |
| 276 | C21orf58 |  |  | 2189 | -0.937 | -0.3149 | Yes |
| 277 | SGO1 |  |  | 2190 | -0.942 | -0.3102 | Yes |
| 278 | CENPH |  |  | 2191 | -0.943 | -0.3055 | Yes |
| 279 | CENPW |  |  | 2192 | -0.943 | -0.3008 | Yes |
| 280 | LMNB1 |  |  | 2194 | -0.950 | -0.2966 | Yes |
| 281 | HJURP |  |  | 2195 | -0.962 | -0.2918 | Yes |
| 282 | UBE2T |  |  | 2196 | -0.965 | -0.2870 | Yes |
| 283 | FANCI |  |  | 2198 | -0.966 | -0.2827 | Yes |
| 284 | CDKN3 |  |  | 2199 | -0.979 | -0.2778 | Yes |
| 285 | CCNB2 |  |  | 2200 | -0.983 | -0.2730 | Yes |
| 286 | CKS1B |  |  | 2201 | -0.987 | -0.2680 | Yes |
| 287 | HIST1H1E |  |  | 2202 | -0.992 | -0.2631 | Yes |
| 288 | SGO2 |  |  | 2203 | -1.003 | -0.2581 | Yes |
| 289 | ESCO2 |  |  | 2204 | -1.004 | -0.2531 | Yes |
| 290 | SMC2 |  |  | 2205 | -1.006 | -0.2481 | Yes |
| 291 | KIF11 |  |  | 2206 | -1.008 | -0.2431 | Yes |
| 292 | EZH2 |  |  | 2207 | -1.017 | -0.2380 | Yes |
| 293 | SPC25 |  |  | 2208 | -1.019 | -0.2330 | Yes |
| 294 | XRCC2 |  |  | 2210 | -1.028 | -0.2284 | Yes |
| 295 | DHFR |  |  | 2212 | -1.040 | -0.2237 | Yes |
| 296 | KNL1 |  |  | 2213 | -1.054 | -0.2185 | Yes |
| 297 | NUDT1 |  |  | 2214 | -1.054 | -0.2132 | Yes |
| 298 | MYBL2 |  |  | 2215 | -1.058 | -0.2080 | Yes |
| 299 | CLSPN |  |  | 2216 | -1.060 | -0.2027 | Yes |
| 300 | CENPN |  |  | 2217 | -1.079 | -0.1973 | Yes |
| 301 | UBE2C |  |  | 2218 | -1.081 | -0.1919 | Yes |
| 302 | CENPU |  |  | 2219 | -1.088 | -0.1865 | Yes |
| 303 | NCAPG |  |  | 2222 | -1.122 | -0.1820 | Yes |
| 304 | H2AFX |  |  | 2223 | -1.146 | -0.1763 | Yes |
| 305 | CDK1 |  |  | 2225 | -1.153 | -0.1711 | Yes |
| 306 | PTTG1 |  |  | 2226 | -1.198 | -0.1651 | Yes |
| 307 | MAD2L1 |  |  | 2228 | -1.204 | -0.1596 | Yes |
| 308 | UBE2S |  |  | 2229 | -1.205 | -0.1536 | Yes |
| 309 | GTSE1 |  |  | 2230 | -1.220 | -0.1476 | Yes |
| 310 | DBF4 |  |  | 2232 | -1.247 | -0.1419 | Yes |
| 311 | RRM2 |  |  | 2234 | -1.268 | -0.1361 | Yes |
| 312 | SMC4 |  |  | 2236 | -1.281 | -0.1302 | Yes |
| 313 | PBK |  |  | 2237 | -1.325 | -0.1236 | Yes |
| 314 | ATAD2 |  |  | 2238 | -1.381 | -0.1168 | Yes |
| 315 | TMPO |  |  | 2239 | -1.382 | -0.1099 | Yes |
| 316 | PRC1 |  |  | 2240 | -1.397 | -0.1029 | Yes |
| 317 | HMGB2 |  |  | 2241 | -1.469 | -0.0956 | Yes |
| 318 | BIRC5 |  |  | 2242 | -1.469 | -0.0883 | Yes |
| 319 | ASPM |  |  | 2244 | -1.490 | -0.0814 | Yes |
| 320 | PCLAF |  |  | 2245 | -1.516 | -0.0739 | Yes |
| 321 | TOP2A |  |  | 2246 | -1.562 | -0.0661 | Yes |
| 322 | NUSAP1 |  |  | 2247 | -1.600 | -0.0582 | Yes |
| 323 | TPX2 |  |  | 2248 | -1.609 | -0.0502 | Yes |
| 324 | CENPF |  |  | 2249 | -1.691 | -0.0418 | Yes |
| 325 | HIST1H1D |  |  | 2250 | -1.720 | -0.0332 | Yes |
| 326 | HIST1H1B |  |  | 2251 | -1.796 | -0.0243 | Yes |
| 327 | MKI67 |  |  | 2252 | -1.892 | -0.0148 | Yes |
| 328 | HIST1H4C |  |  | 2253 | -2.984 | 0.0000 | Yes |
Table: GSEA details [plain text format]

  

Fig 2: DOROSZKO\_U3054MG\_CLUSTER\_4: Random ES distribution      
 Gene set null distribution of ES for **DOROSZKO\_U3054MG\_CLUSTER\_4**
